# Use of Drugs and Cost of Treatment of Diarrhea in Secondary Level Government Hospitals in Maharashtra

**DOI:** 10.4103/0250-474X.70498

**Published:** 2010

**Authors:** P. H. Rao, S. G. Kabra

**Affiliations:** Administrative Staff College of India, Bella Vista, Khairatabad, Hyderabad - 500 082, India; 1 Santokba Durlabhji Memorial Hospital, 15, Vijayanagar, D Block, Malaviyanagar, Jaipur - 302 017, India

**Keywords:** Cost of treatment, diarrhoea, furazolidone, metronidazole, oral rehydration salts, prescription audit, rational use, secondary level hospitals

## Abstract

A prescription audit was carried out among the outpatient attendees of 31 secondary level hospitals under Maharashtra Health Systems Development Project. Use of drugs and cost of treatment of diarrhoea were studied using the prescriptions for diarrhoea collected for the prescription audit. Average number of drugs prescribed per prescription for treatment of diarrhoea was 3.7. It was higher than average number of drugs per prescription in the Maharashtra Health Systems Development Project hospitals in general. About three fourths of the prescriptions contained oral rehydration salts. Furazolidone and metronidazole were prescribed in about half of the prescriptions. Cotrimoxazole was prescribed in about one fourth of prescriptions. About 60% of the prescriptions contained other drugs. The average cost of prescription for diarrhoea was Rs. 14 and increased with the number of drugs prescribed. Average cost of prescription was the highest for those written by general practitioners. Pathological tests were indicated only in case of 11%.

The World Health Organization (WHO) defines diarrhoea as the passage of unusually loose or watery stools, usually at least three times in a 24 h period. However, it is the consistency of the stools rather than the number that is most important. Frequent passing of formed stools is not diarrhoea[1]. Diarrhoeal diseases account for about 8.2 per cent of the total burden of disease in India, contributing about 22 million of Disability Adjusted Life Years (DALYs) lost, the highest among communicable diseases[2]. According to National Family Health Survey (NFHS)–III[3] about 9 per cent of children under age five in India had diarrhoea in the two weeks preceding the survey. Every 30 seconds a child dies due to diarrhoea globally and diarrhoeal diseases are the major contributor to child deaths, accounting for about 35%[4]. Since, diarrhoeal diseases contribute significantly to both morbidity and mortality it is necessary to analyze the use of drugs and the cost of treatment of diarrhoea in India.

Studies conducted during the 1990s reported low use of Oral Rehydration Salts (ORS) in treatment of diarrhea, 18% in West Bengal[5] ; 22% in Delhi[6] and 30.5% in Bangalore[7]. Use of ORS was found to be comparatively higher among those treated at public facilities (45%) than those treated at private sources (37%)[8]. Some studies reported high levels of prescription of antibiotics/antimicrobials and other drugs[6]. A study conducted in Indore revealed that only in case of 20% the choice of the antidiarrhoeal drug was correct[9].

An analysis of prescriptions of outpatients collected from an all India sample of doctors in private practice by C-Marc revealed that 59% contained fixed dose combinations (FDCs) and contributed to the cost significantly[10]. A study on cost of treatment of diarrhoea among patients admitted to Infectious Diseases Hospital in Pune City[11] estimated the average cost borne by the hospital as Rs. 164.87 and that borne by the patients as Rs. 111.36, a total cost of Rs. 276.23. Another study in Kerala[12] estimated cost of drugs between Rs. 37.86 to Rs. 80.00 and the total cost (including travel and fee) between Rs. 60.57 to 129.50. The higher level of cost was attributed to high levels of inpatient treatment. Increase in cost of treatment with frequent changing of the doctor during the treatment of diarrhoea among under-fives was reported in a study conducted in Uttar Pradesh[13].

A prescription audit was carried out among a representative sample of 31 secondary level hospitals under the Maharashtra Health Systems Development Project (MHSDP). A total of 14,004 prescriptions collected for the purpose of Prescription Audit. This paper is based on prescriptions written for diarrhoea only. An analysis of use of drugs and cost of treatment is presented.

The sample hospitals were selected to cover all the eight administrative regions of the state of Maharashtra. A carbon copy of the ’first encounter prescription’ in a specially designed format was obtained from the out patient departments (OPDs) of the sample hospitals. All the four categories of secondary level hospitals in Maharashtra state viz. a) district hospitals, b) sub district hospitals 100 beds, c) sub district hospitals 50 beds, and d) community health centers from each region were included in the study. A total of 14,004 prescriptions were collected from the OPDs of the sample hospitals. The forms were filled during May-June 2003 by the doctors of MHSDP hospitals selected for the study. The contribution of 8 district hospitals was 43%; that of 7 sub district hospitals with 100 beds 26%; that of 8 sub district hospitals with 50 beds 17% and that of 8 CHC PIs with 30 beds 14%. The diagnoses were classified according to the International Classification of Diseases (ICD)-10 of the WHO. The drugs were classified according to Anatomical Therapeutic Classification of the WHO.

Prescriptions for diarrhoea and gastroenteritis (A09 according to ICD-10) were among the first five in all type of MHSDP hospitals. Details are shown Table 1. For the sake of comparability prescriptions containing only single diagnosis are considered for analysis in the present paper. The following analysis is based on 319 prescriptions with diarrhoea as single diagnosis. Prescriptions with diagnosis as dysentery were not considered in this analysis as they are coded as A06 as per ICD-10.

**TABLE 1:** CONTRIBUTION OF PRESCRIPTIONS FOR DIARRHEA BY TYPE OF HOSPITAL RANK AND SHARE

Type	Rank	Share (%)
District hospitals	4	4.7
Sub district hospitals - 100	3	7.8
Sub district hospitals - 50	3	6.0
Community Health Center	5	6.5

Prescriptions for diarrhoea rank 4 among the diseases diagnosed at district hospitals, based on the prescriptions collected. Prescriptions for diarrohea constitute 4.7% of the total prescriptions contributed by district hospitals. The explanation is similarly for different types of hospitals

About eight percent prescriptions for diarrhoea did not contain any drug. The average size of prescription (excluding zero drug prescriptions) for diarrhoea is 3.7, which is higher than the overall average size 3.1 for all prescriptions in the sample MHSDP hospitals[14]. The average number of drugs per prescription is the highest at the lower level i.e. Community Health Centers (CHCs) (3.9) compared to the hospitals at higher level (3.4).

A total of 63 different drugs were prescribed in treatment of diarrhoea in 292 prescriptions containing at least one drug. Frequently used drugs in the treatment of diarrhoea in different categories of hospitals are shown in Table 2. About 75 per cent of the prescriptions contained ORS. Prescription of ORS is the highest among CHCs (81.7%) and lowest in Sub District Hospitals (SDHs) 50 (71.2%).

**TABLE 2:** FREQUENTLY PRESCRIBED DRUGS IN TREATMENT OF DIARRHOEA BY HOSPITAL CATEGORY

Drug Name	Hospital category				Total
	DH	SDH100	SDH50	CHC	
Oral Rehydration Salt	61	56	52	49	218
Furazolidone	17	53	47	38	155
Metronidazole	45	38	32	31	146
Cotrimoxazole	36	15	10	7	68
Oxytetracycline	7	8	9	9	33
Ciprofl oxacin	11	4	6	4	25
Nalidixic acid	3	0	13	8	24
Tetracycline	1	1	12	9	23
Paracetamol	16	15	5	4	40
Metoclopramide	4	5	5	16	30
Ranitidin	16	3	7	3	29
Others	60	52	46	38	176
Total prescriptions	82	77	73	60	292

DH: District hospital; SDH 100: Sub district hospital 100 beds; SDH 50: Sub district hospital 50 beds; CHC: Community Health Center

About 5% of the prescriptions for diarrhoea contained two or more formulations of antibiotics/antibacterials. Prescription of antibiotics/antibacterials is the lowest in SDH100 (about 45%) and the highest in CHCs (about 67 percent). Furazolidone is the most frequently prescribed drug in this category (53%). Prescription of furazolidone is highest at SDH 100 (68.8%) and lowest at district hospitals (20.7%). Metronidazole, an antiprotozoal drug, is the second most frequently prescribed drug in this category. Proportion of prescriptions with metronidazole is the highest at district hospitals (54.9%) and lowest at SDH50 hospitals (43.8%). Cotrimoxazole is the third most frequently prescribed drug in this group (23.3%). Prescription of cotrimoxazole is the highest at district hospitals (43.9%) and the lowest at CHC-PI (11.7%). Other antibiotics/antibacterials prescribed include oxytetracyclin (11.3%), ciprofloxacin (8.7%), nalidixic acid (8.2%) and tetracycline (7.9%). About 60 per cent of prescriptions contained other drugs. Among them frequently prescribed drugs are, paracetamol (13.7%), metoclopramide (10.3%) and ranitidine (9.9%).

Out of the 292 prescriptions for diarrhoea, cost of prescription could be calculated only in case of 64 prescriptions, where information on all aspects of drug like formulation, strength, dose, number of days and price is available. The average cost of prescription is Rs. 14 with median at Rs. 17.29. The average cost and median are given in Table 3 according to the number of drugs prescribed.

The average cost of the prescriptions for diarrhoea is the highest for those given by General Practitioners (GPs) at Rs. 23.69, closely followed by those given by Obstetric and Gynecologists Rs. 21.0 and by pediatricians Rs. 16.6. Only in case of 11 per cent a test is indicated in the prescription for treatment of diarrhoea.

**TABLE 3:** AVERAGE COST OF PRESCRIPTION FOR DIARRHOEA ACCORDING TO THE NUMBER OF DRUGS PER PRESCRIPTION

Number of drugs per prescription	Number of prescriptions	Cost of prescription	
		Mean (Rs.)	Median (Rs.)
1	6	11.4	10.0
2	19	10.4	11.5
3	29	18.9	16.6
4	8	24.3	20.2
5	2	49.1	49.1

While total number of prescriptions for diarrhoea is 292, cost information could be worked out only for 64 as complete details like dosage, formulation etc. were not available for other prescriptions.

Simple hygiene measures like hand washing before meals and after defecation by children and care givers like mothers, improved sanitation and provision of safe drinking water contribute significantly in reducing the burden due diarrhoeal diseases. However, due to multiple factors morbidity and mortality due to diarrhoeal diseases continue to be high in India. Hence, management of diarrhoeal diseases assumes importance.

Rational use of drugs has two aspects. The first is selecting drugs from the essential drug list (EDL). The second is prescribing them rationally, which involves following the Standard Treatment Guidelines (STGs). Replacement of adequate fluids in any form, especially glucose/ electrolyte drinks (oral rehydration solutions) is the main nonpharmacological intervention preferred in the treatment of diarrhoea. Pharmacological interventions like use of antibiotics/antibacterials are indicated only in specific cases. Ciprofloxacin is indicated in very ill patients and metronidazole in amoebic dysentery[15].

ORS is an essential drug as per the 15^th^ EDL of WHO[16] and EDL of India[17]. Compared to the earlier studies, the present study presents favourable picture in this regard among the secondary level government hospitals in Maharashtra. This could be due to sustained efforts by various bodies to increase awareness among physicians during the recent times.

All the major antibiotics/antibacterials prescribed in the MHSDP sample hospitals, except for oxytetracyclin are included in the national EDL in India. However, antibiotics/antibacterials have limited role in treatment of diarrhoea and their use justified only in cases where specific bacterial/protozoal infection is identified/suspected. Unnecessary use of antibiotics/antimicrobials and other drugs not only increase the cost of treatment but also may prove harmful or counter productive. Need for and selection of antibiotics/antibacterials in treatment of diarrhoea will be appropriate if done based on examination of the stools. High use of furazolidone (53%), metronidazole (50%) and cotrimoxazole (23.3%) found in this study is questionable as only in case of 11% a test was indicated before prescribing drugs.

Paracetamol (antipyretic/analgesic), metoclopromide (antiemetic) and ranitidin (antiulcerant) are all essential drugs according to the national EDL. Their use, in treatment of diarrhoea in the MHSDP hospitals could have been necessitated by the presence of symptoms like fever, vomiting and acidity along with diarrhoea. However, rationale for the presence of other drugs in almost 60 per cent of the prescriptions for diarrhoea is difficult to explain.

The study found that cost of treatment goes up with the average number of drugs per prescription from Rs. 11.40 (one drug) to Rs. 49.10 (five drugs). It was also observed that average number of drugs per prescription in case of diarrhoea is higher than the average number of drugs per prescription in case of all diagnoses together.

To encourage rational use of drugs and cost effective treatment of diarrhoea, there is a need to create an enabling environment. The first step in creating an enabling environment is to have an EDL and STGs for management of diarrhoea. Prescribing only those drugs that are included in the EDL of the state (EDL of India or EDL of WHO, in case a state does not have an EDL) is the key to limit the cost of drugs. It is also equally essential that the drugs selected are from an EDL are prescribed rationally by following the STGs for diarrhoea, developed by the state or national agencies. This will limit use of antibiotics/antibacterials and other drugs, thus keeping the cost of treatment of diarrhoea manageable. ORS should be an essential component in the management of diarrhoea, especially among the children to avoid deaths due to dehydration. The second step in creating an enabling environment is promoting awareness about rational use of drugs, concept of essential drugs and STGs among the prescribers. The third step is to improve accessibility to STGs to the doctors.

Ensuring mechanisms like continuous prescription audits to monitor and improve the prescribing habits of doctors need to be put in place to ensure that the doctors adhere to the EDL and follow the STGs. Proper use of STGs have been effective in reducing use of drugs for diarrhoeal diseases and increasing use of ORS in India[18].

The Government of Orissa piloted path-breaking initiatives in this regard. The initiative includes measures that include, making the doctors accountable for the drugs prescribed in case of five diseases, including diarrhea, which account for a major part of the state’s disease burden and reimbursement of expenditure of drugs to patients by Chief District Medical Officer (CDMO) if the drugs included in the protocol are out of stock or drugs not in the protocol are prescribed[19]. The pilot project could not be implemented for long and tested for their effectiveness but the Government of Orissa is keen on reviving it[20].

Rational use of drugs can also be improved if pathological tests are used for identifying causative microorganism, to decide on the necessity of prescribing an antibiotic/antibacterial, which improves the effectiveness of treatment.
